# Death associated protein kinase 2 is expressed in cortical interstitial cells of the mouse kidney

**DOI:** 10.1186/1756-0500-7-345

**Published:** 2014-06-07

**Authors:** Justin A Guay, Don M Wojchowski, Jing Fang, Leif Oxburgh

**Affiliations:** 1 Center for Molecular Medicine, Maine Medical Center Research Institute, 81 Research Drive, Scarborough, ME 04074, USA

**Keywords:** DAPK2, Kidney, Chronic kidney disease, Fibrosis, TGFβ, Cisplatin

## Abstract

**Background:**

DAPK2 is a pro-apoptotic protein kinase that associates with TGFβ receptors. The homolog DAPK1 has been shown to mediate apoptosis in kidney injury. Expression databases indicate that DAPK2 is expressed in the kidney, and in this work we investigate the localization of renal DAPK2 expression and its role in the kidney.

**Results:**

Immunostaining demonstrates DAPK2 expression in interstitial cells of the renal cortex including PDGFRβ-positive pericytes and the CD73-positive erythropoietin-expressing fibroblast population. Tubulointerstitial fibrosis in experimental CKD arises directly from resident interstitial cells, and we therefore evaluated the expression of DAPK2 in the expanded interstitium of mice with kidney disease induced by chronic cisplatin administration. Expanded renal interstitium in these animals was negative for DAPK2 expression, but healthy areas of the kidney in which the tubular interstitium had not expanded expressed DAPK2 at levels similar to the uninjured control. *Dapk2* null mice were generated to evaluate if DAPK2 is required for formation of the kidney, or its maintenance in the adult. Kidneys of *Dapk2* null mice did not show overt malformations or age-related degeneration, but did show a slight increase in the number of interstitial fibroblasts. Differences were seen between *Dapk2* null mice and wild type controls in the response to tubulointerstitial fibrosis caused by chronic cisplatin administration. Although mutant and wild type mice displayed comparable levels of alpha smooth muscle actin, interstitial proliferation and SMAD2 signaling, *Dapk2* null mice showed reduced interstitial collagen accumulation.

**Conclusions:**

In the kidney, DAPK2 is strongly and specifically expressed in interstitial cells of the cortex, providing a useful marker for this important cell population. *Dapk2* null mice are phenotypically normal under steady state conditions, but display some resistance to extracellular matrix deposition in experimental renal fibrosis indicating that DAPK2 plays a profibrotic role in kidney injury.

## Background

Death Associated Protein Kinases (DAPKs) are of interest in kidney disease because they have been associated with both TGFβ signaling and apoptosis [1-6]. The founding member of the family, DAPK1, is a mediator of renal tubular injury in ischemic and obstructive injury models [1-4]. DAPK1 is activated by signals including TGFβ, which plays multiple roles in chronic kidney disease (CKD) [6]. In mouse renal tubule epithelial cells, where it is specifically expressed, DAPK1 mediates apoptosis through activation of p53 [1-5].

The role of the related protein DAPK2 in kidney injury has yet to be determined. DAPK2 is the smallest of the DAPK family members at ~42 kDa. DAPK2 contains a kinase domain with 80% identity to that of DAPK1 and also shares a calmodulin-binding domain with DAPK1. Unlike DAPK1, DAPK2 possesses no cytoskeletal binding domains or other characteristic protein binding domains, and instead contains a homodimerization domain that facilitates the activation of the protein kinase domain [7,8]. *Dapk2* is involved in autophagy and membrane blebbing both with and without complete apoptosis [7].

*In vitro*, DAPK2 responds to cellular stress signaling induced by TNFα and Fas by inducing apoptosis [9]. *Dapk2* is downregulated by Wnt/β-catenin and this downregulation is associated with resistance to apoptosis induced by loss of cell adhesion (anoikis) [10]. *Dapk2* is transcriptionally regulated by E2F1, KLF6, and SP1, a group of factors that coordinately regulate the cell cycle, apoptosis and cancer progression [11]. DAPK2 was identified as a binding partner of TGFβ receptor I in a protein binding assay, although this association has yet to be functionally validated [12].

Public databases report expression of *Dapk2* transcript in several organs including kidney, lung, skeletal muscle, colon, breast, and spleen. DAPK2 is also expressed in leukocytes and erythroblasts where it is involved in their differentiation [13,14]. Considering the intriguing finding that *Dapk2* is expressed in the adult kidney, and is associated with TGFβ signaling, we set out to clarify the localization of DAPK2 protein expression in the kidney and to ascertain if *Dapk2* is required for renal development or maintenance. We find that DAPK2 protein is expressed in interstitial cells of the adult kidney including PDGFRβ-positive pericytes and CD73-positive fibroblasts. Functional analysis suggests that while *Dapk2* does not regulate interstitial proliferation or smooth muscle actin expression in CKD, it does promote extracellular matrix deposition.

## Results and discussion

### Dapk2 loss of function mouse strain

**Figure 1:**
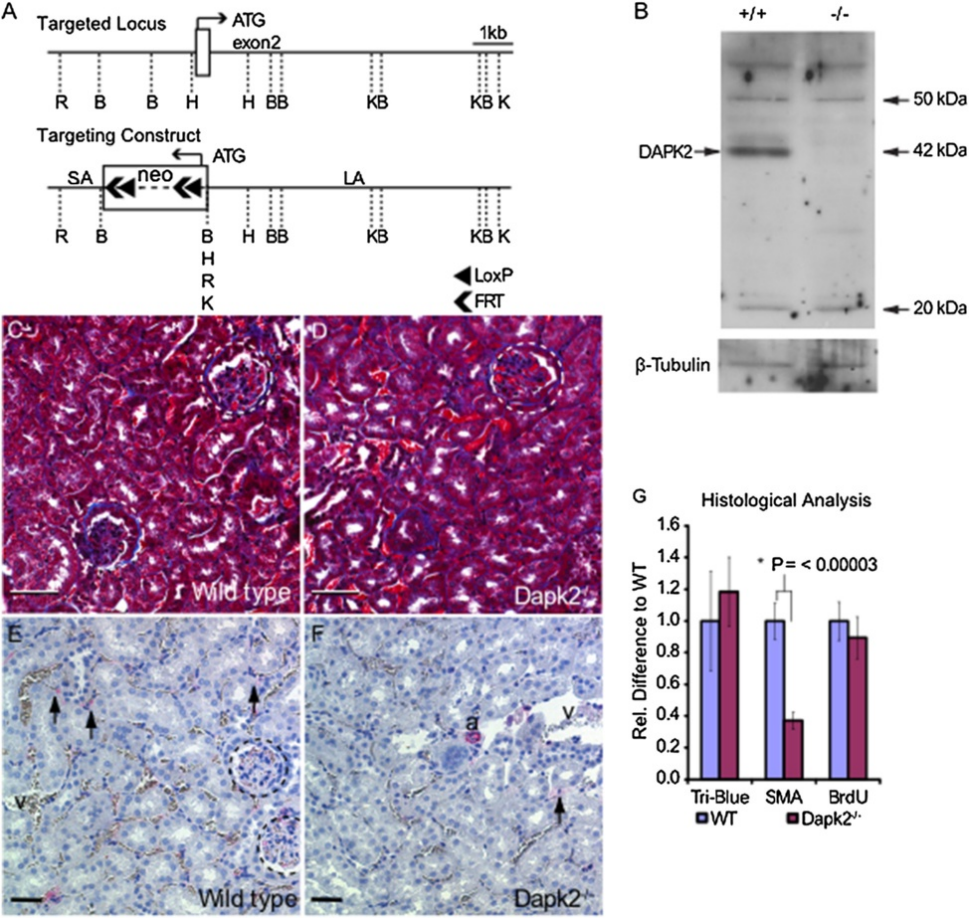
***Dapk2 *null mice possess fewer αSMA expressing interstitial cells. (A)** Exon 2, containing the translation start site, was replaced by a PGK:NEO cassette with internal LoxP and FRT sites. **(B)** Western blotting using a polyclonal c-terminal specific antibody demonstrates that the null mouse does not express DAPK2 at the protein level in kidney. Representative Masson’s Trichrome staining from six **(C)** wild type and **(D)** six *Dapk2* null kidney sections show no gross histological differences between genotypes. **(E-F)** Representative αSMA staining (black arrows) showing fewer positive interstitial cells in mutant mice. **(G)** Graph depicting the relative levels of staining between mice of both genotypes normalized to wild type values. Quantitative analysis is based on 15 fields in untreated mice. Scale bars = 50 μM. Dashed circles mark glomeruli. Abbreviations: B, BamHI; H, HindIII; K, KpnI; R, EcoRI; LA, long arm of targeting construct; SA, short arm of targeting construct.

To determine the localization and role of *Dapk2 in vivo* we developed a *Dapk2* null mouse strain. In this strain, the initiation codon and first portion of the kinase domain are deleted by replacement of exon 2 with a PGK:Neo cassette (Figure 1A). Western blot analysis using a polyclonal antibody raised to the C-terminus of the DAPK2 protein confirms loss of DAPK2 expression in the kidney (Figure 1B). *Dapk2* null mice were bred as a homozygous strain and displayed no overt abnormalities. Histological analysis of Masson’s Trichrome staining (Figure 1C-D), alpha smooth muscle actin (αSMA) staining (Figure 1E,F) and bromodeoxyuridine (BrdU) incorporation (not shown) showed no differences in kidney morphology, proliferation or collagen deposition in *Dapk2* null mice compared to wild type controls, but a reduction in the area of αSMA staining in *Dapk2* null mice (Figure 1G). This reduction is seen largely in the renal interstitium, suggesting a difference in fibroblast or vascular mural cell abundance or expression of αSMA. In counts of CD73 positive fibroblasts cells in two histological sections of three individuals from each genotype we found a total of 114 positive cells in wild types and 159 positive cells in *Dapk2* null supporting a skewing of interstitial cell populations in the null.

### DAPK2 is expressed in a subset of renal interstitial cells

A survey of the Gene Expression Omnibus (GEO) database reveals expression of *Dapk2* transcript in numerous tissues (http://www.ncbi.nlm.nih.gov/sites/entrez?db=geo&term=dapk2). The publicly available Genito-Urinary Development Molecular Anatomy Project (GUDMAP.org) repository identifies several subsets of embryonic and adult kidney cell types in which *Dapk2* is expressed [15]. These include small blood vessels and podocytes in the embryo, and the renal capsule, mesangium, mesangial capillaries, and medullary vasculature in the adult.

**Figure 2:**
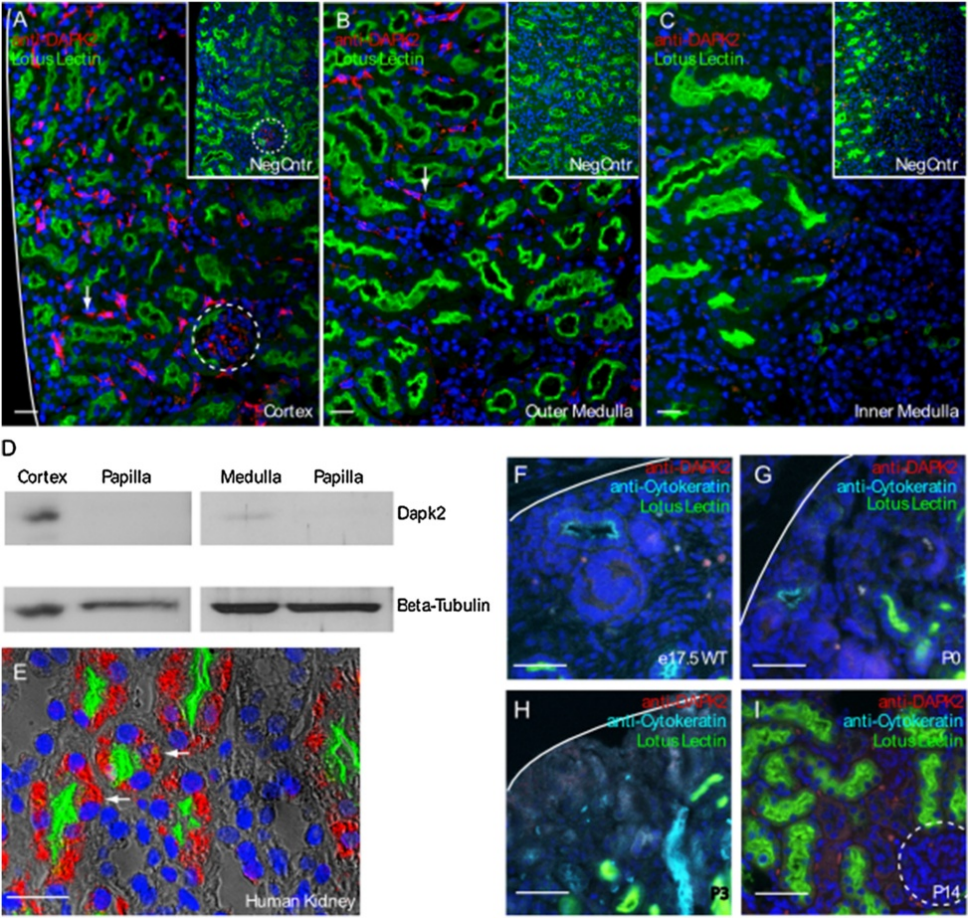
**Cortical peritubular expression of DAPK2 in kidney. (A-C)** Representative immunofluorescent images of DAPK2 staining (white arrows) of six wild type mice. Kidney sections from six *Dapk2* null mice were used as specificity controls for the antiserum (insets A-C). Dashed circles indicate nonspecific staining in glomeruli. DAPK2 was localized to a population of interstitial cells that extend from **(A)** the renal cortex to **(B)** the cortical-medullary boundary. **(C)** No expression of DAPK2 was found in the inner medulla. **(D)** Western Blot analysis of tissue samples from regions of the kidney confirms expression of DAPK2 specifically in the cortex. **(E)** Immunofluorescence of DAPK2 using the same antibody in human kidney shows specific staining in proximal tubules (white arrows). Immunofluroescence images of DAPK2 staining at embryonic and postnatal stages **(F)** E15.5, **(G)** P0, **(H)** P3, and **(I)** P14. Expression is seen only at P14 (arrow). Scale bars = 50 μm.

To specifically localize DAPK2 expression within the kidney, we performed immunofluorescent staining of kidney sections using a N-terminal specific monoclonal antibody. Specificity of the anti-DAPK2 antibody was validated using tissue sections from the *Dapk2* null mouse**.** Very weak background staining can be detected in some glomeruli in the null control tissue precluding definitive confirmation of glomerular *Dapk2* expression (Figure 2A-C, insets). Strong and specific signal is seen in the interstitium of wild type tissue under the same conditions, indicating that DAPK2 is expressed in cells located within the peritubular space.

DAPK2 localizes with a high degree of specificity to a subset of cortical peritubular interstitial cells that are most abundant at the outer edge of the renal cortex between the capsule and the most cortical glomeruli and extends in a diminishing gradient to the cortical-medullary boundary (Figure 2A-C). This observation was supported by western blot of tissue isolated from distinct regions of the kidney (Figure 2D). In contrast to the interstitial expression seen in mouse, human kidney tissue displays DAPK2 expression in epithelial cells of Lotus Lectin expressing proximal tubules (Figure 2E). This is an interesting difference because it implies that DAPK1 and DAPK2 are co-expressed in the proximal tubule of the human kidney [5]. Analysis of embryonic and postnatal stages shows no DAPK2 expression at E15.5, P0, or P3, but sporadic and weak expression in interstitial cells at P14 (Figure 2F-I).

**Figure 3:**
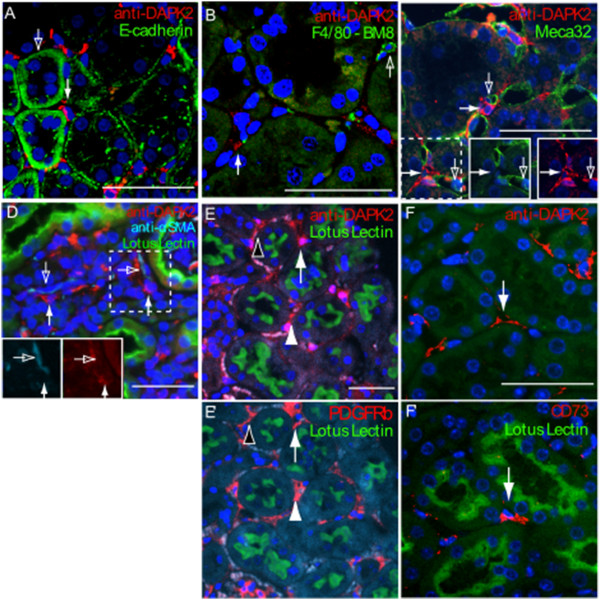
**DAPK2 is expressed in cortical peritubular fibroblasts.** Co-immunofluorescent staining with molecular markers excluded DAPK2 expression (white arrows) from three major cell types. Proximal and distal tubules were excluded via (Figure 2A-C) lotus lectin and **(A)** E-cadherin staining (open arrow), **(B)** dendritic cells and macrophage positive for F4/80-BM8 (open arrow) did not co-localize with DAPK2, and **(C)** endothelial cells marked with membrane localized Meca32 (open arrow) were close to but did not co-localize with DAPK2 (inset green and red channels of dashed box area). **(D)** Smooth muscle cells marked by αSMA did not co-localize with DAPK2 staining (inset: cyan and red channels of dashed box area). Adjacent staining with the pericyte and fibroblast specific marker **(E,E’)** PDGFRβ and **(F,F’)** CD73 respectively, correlated positively with DAPK2 antibody labeling. Immunofluorescent images are representative of three wild type mice analyzed for each antibody. White arrows, white arrowheads, and black arrowheads correspond with equivalent positions between panels and adjacent ‘prime’ panels. Scale bars = 50 μm.

To further characterize the DAPK2 expressing cell population in mouse, co-localization studies using markers for prevalent cell types within the renal interstitium were performed. Staining with proximal tubule specific Lotus Lectin (Figure 2A-C) and distal tubule specific E-cadherin [16-18] (Figure 3A) confirmed that DAPK2 expressing cells reside outside the nephron. Co-staining with the antibody F4/80-BM8 demonstrated that DAPK2 expression does not co-localize with the network of dendritic cells or with macrophages within the kidney [19,20] (Figure 3B). MECA32 (PAL-E/anti-PV-1) a membrane associated vascular-endothelial marker [21-24] did not overlap with cytoplasmic DAPK2 (Figure 3C and insets), indicating that DAPK2 is not expressed in vascular endothelial cells. Because of the proximity of DAPK2 to endothelial cells, we next looked to see if DAPK2 was expressed in pericytes using an antibody specific for PDGFRβ. DAPK2 and PDGFRβ antibodies were both of the same species and not useful for co-staining. We therefore stained adjacent sections for DAPK2 and PDGFRβ respectively and assessed the correlation of expression between the two proteins. Interestingly, we found a strong correlation of DAPK2 expressing cells (Figure 3E) with the much more numerous PDGFRβ expressing pericytes (Figure 3E’). The correlated domains of expression demonstrate that DAPK2 is expressed by renal pericytes.

To test the possibility that DAPK2 is expressed in renal interstitial fibroblasts, we compared DAPK2 staining with CD73, which is expressed in interstitial fibroblasts throughout the cortex [25]. DAPK2 and CD73 antibodies are also not compatible for co-staining, necessitating immunostaining of adjacent sections. Interestingly, adjacent section staining for DAPK2 (Figure 3F) and CD73 (Figure 3F’) revealed a strong correlation of localization. CD73 expression within the renal interstitium correlated with DAPK2 expression in adjacent sections, while many of the more numerous DAPK2 positive cells did not express CD73. DAPK2 may thus be a broad marker of cortical renal interstitial fibroblasts and pericytes that encompasses the CD73 expressing population. CD73 is a well established marker of the erythropoietin expressing cells of the deep cortex of the kidney [26]. The association of DAPK2 and CD73 therefore implies that DAPK2 is expressed in the erythropoietin expressing fibroblast population.

Identification of the DAPK2 expressing cells as peritubular fibroblasts was attempted using anti-fibroblast specific protein 1 (FSP1). Co-staining with anti-DAPK2 and anti-FSP1 showed no co-localization (data not shown). However, FSP1 expression was found in the medulla, in cells that morphologically resembled polymorphonuclear cells as described by others [27,28].

### DAPK2 expression is lost in the expanded tubular interstitium of the fibrotic kidney

**Figure 4:**
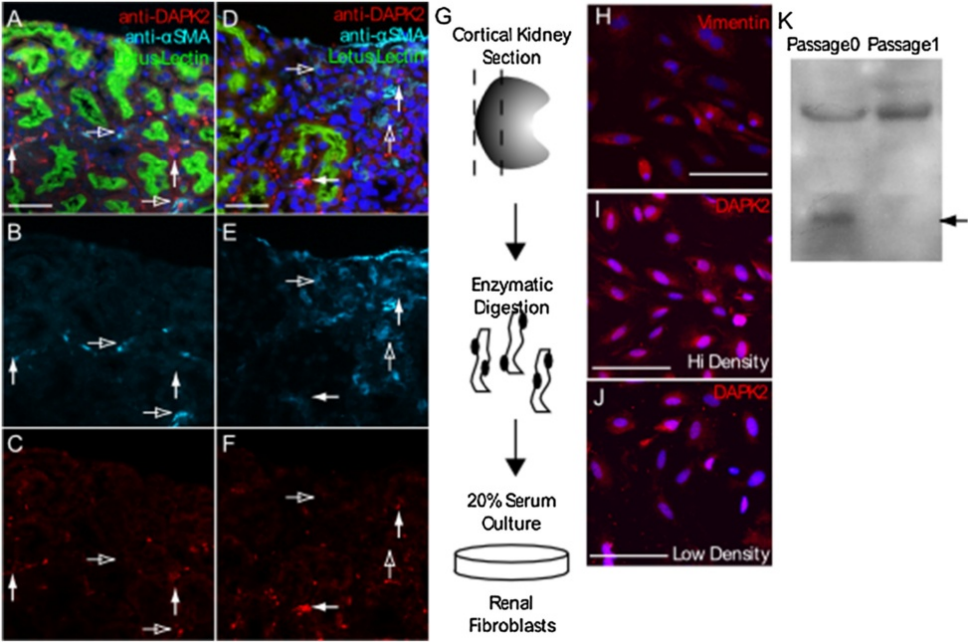
**DAPK2 is not expressed in fibrotic cells.** Representative immunofluorescence of kidney tissue from ten fields analyzed in two wild type **(A-C)** uninjured and **(D-F)** chronic cisplatin injured mice shows no DAPK2 staining in the expanded αSMA positive interstitium of injured mice, white arrows indicate DAPK2 positive cells, open arrows indicate αSMA positive cells. **(G)** Schematic of primary fibroblast isolation. **(H)** Representative immunofluorescent staining of ten fields of cultured primary fibroblasts for the fibroblast marker vimentin in primary cells at passage 0. Representative immunofluorescent staining of ten fields of cultured primary fibroblasts for DAPK2 in **(I)** high and **(J)** low density primary cells at passage 0. **(K)** Immunoblot of DAPK2 expression in isolated real fibroblasts demonstrating loss expression of DAPK2 after expansion and passage, arrow indicates DAPK2 band, higher band shows β - Tubulin loading control. Scale bars = 50 μm.

The majority of myofibroblast accumulation in experimental CKD is derived from resident cells of the peritubular space, which led us to ask if DAPK2 expression associates with expanded fibrotic interstitium [29,30]. To answer this question we evaluated DAPK2 expression in kidneys subjected to seven weeks of chronic cisplatin administration. Chronic cisplatin treatment induces increased collagen deposition across the kidney with little morphologically detectable damage to the nephron epithelium [31] and results in a strong fibrotic response with significant expansion of resident interstitial cells that express αSMA [29,30]. Wild type mice were treated with 3 mg/kg cisplatin seven times at seven day intervals and harvested 52 days after the first injection. Compared to untreated controls (Figure 4A-C), DAPK2 expression in cisplatin treated kidneys was not seen in regions in which the interstitium was expanded, but was instead seen in morphologically normal interstitium surrounding nephrons containing Lotus Lectin positive brush borders (Figure 4D-F). Based on these findings we hypothesized that DAPK2 expression may be downregulated in the process of interstitial cell proliferation. To support this hypothesis, we cultured primary renal fibroblasts derived from the outer renal cortex (Figure 4G). These primary cells were positive for Vimentin and expressed DAPK2 (Figure 4H-J). DAPK2 was not differentially expressed in closely associated high-density cells or in sparse, low-density cells (Figure 4I,J). We next monitored DAPK2 expression in confluent cells by immunoblot. Following outgrowth of isolated cells, DAPK2 could readily be detected by immunoblotting. However, after a single passage DAPK2 could no longer be detected, suggesting that DAPK2 expression may either be down regulated in cultured cells, or that DAPK2 expressing cells suffer a proliferative disadvantage and are out competed in culture (Figure 4K).

### Loss of Dapk2 influences the fibrotic response to chronic cisplatin treatment

**Figure 5:**
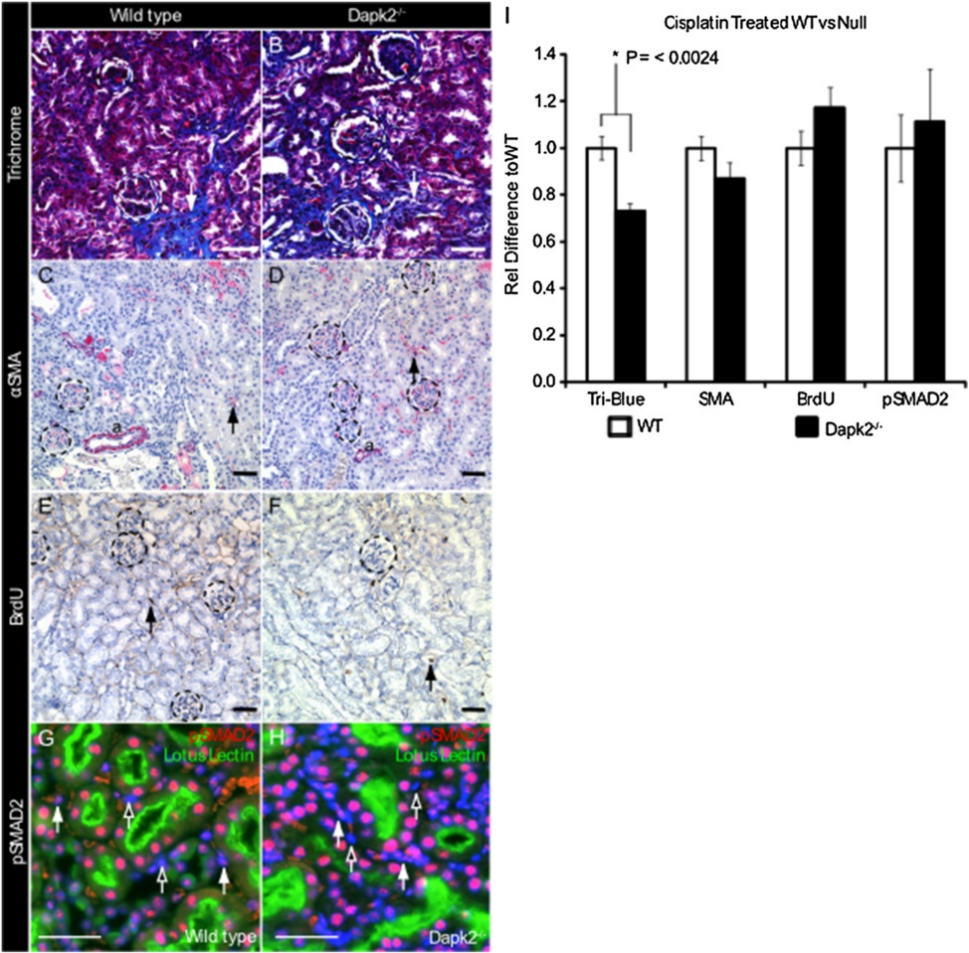
***Dapk2 *null mice show resistance to fibrosis.** In a chronic cisplatin induced model of CKD, 12 wild type and 10 mutant animals were analyzed for markers of fibrosis. Representative images show **(A,B)** reduced collagen deposition in the *Dapk2* null as demonstrated by representative Masson’s trichrome staining (blue color indicated by arrows), and similar levels of **(C,D)** αSMA and **(E,F)** BrdU staining after treatment (arrows indicate positive cells, ‘a’ indicates arterial smooth muscle). **(G,H)** pSMAD2 staining after treatment showing equivalent numbers of positive interstitial nuclei, white arrows indicate positive pSMAD2 interstitial nuclei, open arrows indicate pSMAD2 negative nuclei. **(I)** Graph depicting the relative levels of staining between cisplatin treated of both genotypes normalized to untreated wild type values. Quantitative analysis is based on 100 fields in cisplatin treated mice. Dashed circles = glomeruli, scale bars = 50 μM.

**Figure 6:**
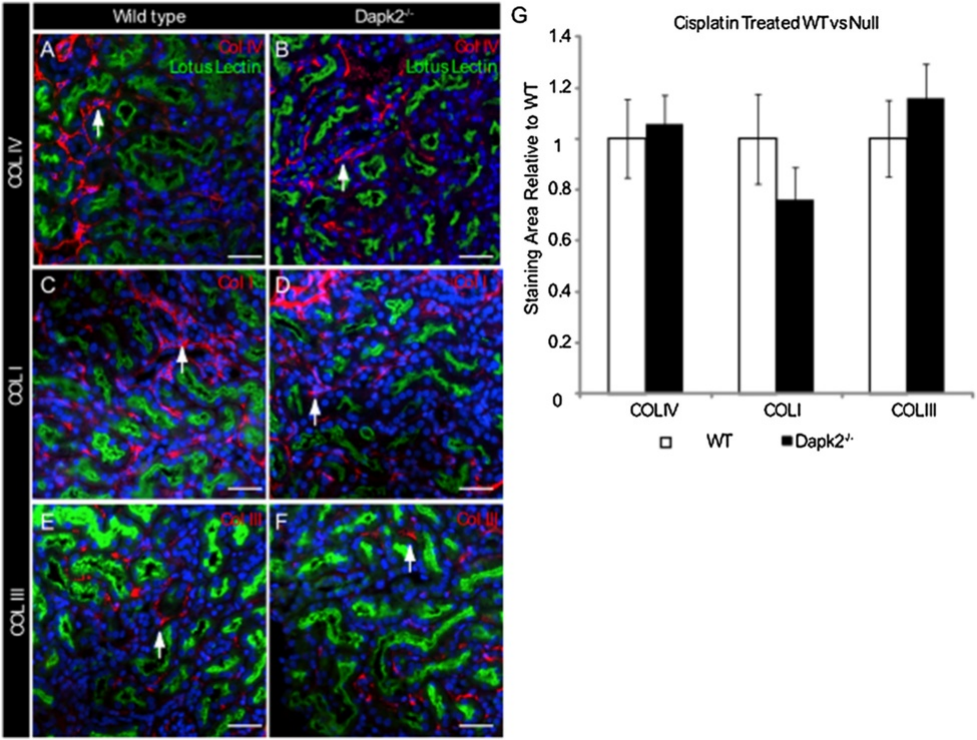
**Immunostaining for specific collagens.** Immunofluorescent staining of wild type and *Dapk2* null mice for **(A,B)** Collagen IV, **(C,D)** Collagen I, and **(E,F)** Collagen III showing a trend toward decreased deposition of Collagen I only. Arrow show examples of positive staining for each collagen. **(G)** Graph showing the relative collagen staining area from at least 54, 40× fields per genotype. Scale bars = 50 μM

To evaluate the role of *Dapk2* in CKD we next treated 12 wild type and 12 *Dapk2* null mice with chronic cisplatin injections as described above. Compared to untreated controls, both genotypes showed increases in collagen deposition (blue stain in Masson’s trichrome), αSMA expression, and proliferation (BrdU incorporation). A significant difference could be seen in trichrome staining (Figure 5A,B), with *Dapk2* null kidneys displaying approximately 20% less collagen deposition (Figure 5I). However, levels of αSMA (Figure 5C-D) and proliferation (Figure 5E,F) were comparable between wild type and null injured mice. Because DAPKs act as modulators of TGFβ signaling in other systems, we also evaluated activation of the TGFβ pathway by immunostaining for phosphorylated SMAD2. However, no difference could be detected between wild type and null injured mice (Figure 5G,H). Collagen deposition is a key feature of tubulointerstitial fibrosis, and the finding that *Dapk2* null kidneys are moderately protected suggests that DAPK2 may play a role directly in the collagen deposition pathway. However, the role of DAPK2 in this process appears to be downstream of TGFβ mediated SMAD signaling because the level of interstitial SMAD2 signaling is not significantly different between the two genotypes.To further assess the observed difference in collagen staining seen via Masson’s Trichrome we stained chronic cisplatin injured kidneys for Collagens IV, I, and III. (Figure 6A-F). In our analysis of at least nine mice and 54 40× images for each genotype we found no significant difference in Collagen IV or Collagen III staining area, but a trend toward reduction in Collagen I staining area (Figure 6G).

## Conclusion

In this study we have shown that DAPK2 is expressed in peritubular interstitial cells of the renal cortex including the PDGFRβ expressing pericyte and CD73 expressing renal fibroblast populations in the mouse. In contrast to the mouse, DAPK2 is expressed in epithelial cells of the nephron in the human kidney, where published studies indicate DAPK1 is also expressed [5]. This has important implications for the translation of kinase specific inhibition or activation between mice and humans. Adult *Dapk2* null mice have morphologically normal kidneys, but when challenged with chronic cisplatin administration they display a decreased propensity for extracellular matrix deposition in the interstitial space. While there was no statistical significance in collagen staining in injured mice, Collagen I trended toward a decreased staining area in the *Dapk2* null. This suggests that some combination of differentially expressed collagens may be responsible for the difference in gross collagen area observed between wild type and mutants. Based on these observations we conclude that DAPK2 promotes tubulointerstitial fibrosis in the mouse.

## Methods

### Animals

Animal care in accordance with the National Research Council Guide for the Care and Use of Laboratory Animals was approved by the Maine Medical Center IACUC. Except where indicated all animals used were 6–8 weeks of age. *Dapk2* null mice were generated as in [32]. *Dapk2* genomic clones were isolated from a 129svJ λ phage library by hybridization to a *Dapk2* cDNA. A targeting vector containing a PGK:Neo selection cassette was transfected into ES cells and cells were subjected to G418 drug selection and cloned. Homologous recombination was detected by southern blotting and targeted cells were injected into 129SV/J blastocysts. Progeny of chimeras were bred with 129SV/J mice for five generations before analysis. *Dapk2*^+^/-mice were intercrossed to yield *Dapk2* null mice. The *Dapk2* null strain was maintained on a 129SV/J background and wild type controls were 129SV/J. 12 wild type and 10 mutant mice were intraperitoneally injected with 3 mg/kg cisplatin seven times at 7 day intervals. Kidneys were harvested at 52 days after the first injection. Treated animals were analyzed relative to four untreated mice of both genotypes.

### Primary cell isolation

Dissected kidneys were cut sagittally and the cortical region of the kidney was separated. Cortical sections were placed onto tissue culture plates coated with 1% gelatin. Minced kidney bits were spread across the gelatin plate by scratching with a razor blade. After air-drying for 5 min at 37°C fibroblast enrichment medium (20% Bovine Growth Serum, DMEM) with Penicillin-Streptomycin, Amphotericin B was added and fibroblasts were permitted to grow. Cells were passaged at confluency to 1% gelatin coated plates.

### Immunostaining

Tissue was fixed in 4% paraformaldehyde for 1 hour per millimeter thickness at room temperature before paraffin sectioning. Immunohistochemical samples were treated for 10 minutes in 1% H2O2. All antibody assays except before anti-CD73 were treated by antigen unmasking using citrate buffer (Dako Cytomation, 1:10 dilution) and boiling for 10 minutes. Primary antibody concentrations: 1:50 α-smooth muscle actin (Invitrogen); 1:25 CD73 (BD Biosciences); 1:50 Collagen I (Rockland); 1:50 Collagen III (Rockland); 1:50 Collagen IV (Rockland); 1:50 DAPK2 (Epitomics); 1:100 F4/80–BM8 (Santa Cruz Biotech); 1:200 Lotus Lectin (Vector Labs); 1:50 MECA32 (Developmental Studies Hybridoma Bank; 1:50 PDGFRβ (Epitomics). Secondary antibodies: 1:200 goat anti-rabbit Alexa568 (Invitrogen); 1:200 mouse anti-rat biotin (Jackson Immunoresearch); 1:200 Avidin-488 (Invitrogen). TUNEL staining was performed using the Apoptag® Peroxidase in situ kit (Millipore) according to the manufacturer’s protocol. Biotinylated secondary antibody incubation was done in PBS at Room Temperature for 45 minutes, followed by R.T.U ABC Elite Reagent incubation at Room Temperature for 30 minutes. Sections were then visualized using Sigma Fast 3,3’-diaminobenzidine (Sigma-Aldrich). After color development sections were counterstained with Meyers hematoxylin, coverslipped and mounted with Cytoseal 60 (Richard-Allan Scientific).

### Quantitative analysis of histochemistry

Images (.tif) were cropped and analyzed by Color Threshold for area or nuclei count using ImageJ. Values were normalized to the total image area minus the area of white space. 15 histological sections from a total of four mice were analyzed for each genotype of untreated control mice. 100 histological sections from a total of seven mice were analyzed for each genotype in Cisplatin treated mice. Trichrome analysis was performed at 40×, while αSMA and BrdU analysis was carried out at 20×.

### Immunoblot

Analysis was performed on 10% acrylamide gels, transferred onto nitrocellulose membranes, blocked in 5% BSA for 30 min and incubated in primary antibody over night at 4°C. Primary antibody concentrations: 1:5,000 DAPK2 (Chemicon) and 1:5,000 β-tubulin (Santa Cruz Biotech). Secondary antibody concentrations: 1:40,000 anti-rabbit-HRP (Jackson Immunological). Blots were developed using HyGLO chemiluminescent HRP antibody detection regent (Denville Scientific).

### Availability of supporting data

No supporting data.

## Abbreviations

DAPK: Death associated protein kinase; CKD: Chronic kidney disease; GEO: Gene expression omnibus; GUDMAP: Genito-urinary development molecular anatomy project; FSP1: Fibroblast specific protein 1; αSMA: Alpha smooth muscle actin; BrdU: Bromodeoxyuridine.

## Competing interests

The authors declare that they have no competing interests, financial or otherwise.

## Authors’ contributions

JAG designed study, performed experiments and wrote manuscript; DMW designed study; JF designed study and performed experiments; LO designed study, performed experiments and wrote manuscript. All authors read and approved the final manuscript.
